# Prediction of thyroidal ^131^I effective half-life in patients with Graves' disease

**DOI:** 10.18632/oncotarget.20849

**Published:** 2017-09-12

**Authors:** Ruiguo Zhang, Guizhi Zhang, Renfei Wang, Jian Tan, Yajing He, Zhaowei Meng

**Affiliations:** ^1^ Department of Nuclear Medicine, Tianjin Medical University General Hospital, Tianjin, China

**Keywords:** Graves’ disease, effective half-life, radioiodine therapy, iodine uptake, predictive model

## Abstract

**Purpose:**

Calculation of effective thyroidal half-life (Teff) of iodine-131(^131^I) is cumbersome and tedious. The aim of this study was to investigate factors that could be used to predict Teff and to develop a Teff prediction model in Graves’ disease patients.

**Methods:**

A total of 256 patients with GD were involved in this study. We investigated the influences of age, gender, disease duration, thyroid weight, antithyroid drugs, antithyroid drugs discontinuation period (ADP), thyroid function indexes, thyroid autoantibodies, thyroid-stimulating hormone receptor antibody (TRAb) level and radioactive iodine uptake (RAIU) values before ^131^I therapy on Teff, applying univariate and multivariate analyses.

**Results:**

Teff correlated negatively with thyroid peroxidase antibody, TRAb and thyroid weight, as well as positively with 24-hour, 48-hour, and 72-hour RAIU. Additionally, a longer ADP (especially≥ 14d) or without antithyroid drugs before ^131^I therapy led to a longer Teff. Stepwise multiple linear regression analysis showed that 24-hour and 72-hour RAIU were statistically significant predictors of Teff (*P*<0.001). The relationship was: predictive Teff=5.277+0.295×72-hour RAIU-0.217×24-hour RAIU (r =0.865, *P* < 0.001).

**Conclusion:**

The present results indicate that prediction of Teff from 24-hour and 72-hour RAIU is feasible in patients with Graves’ disease, with high prediction accuracy.

## INTRODUCTION

Graves’ disease (GD) is an autoimmune disorder in which thyroid-stimulating hormone receptor antibodies cause the thyroid gland to synthesize and release large amounts of thyroid hormones [1, 2]. Radioiodine-131 (^131^I) is increasingly used as the treatment of choice in most patients with Graves’ hyperthyroidism and has been proven to be efficient and safe both as a primary therapy and secondary therapy when thyrotoxicosis can not be controlled by antithyroid drugs (ATD) [3, 4].

The dose of ^131^I to be administered could be fixed even if neither thyroid weight nor thyroid uptake is known and also adjusted using complex dosage formula [5–7]. Two types of dosage formulas are usually used in clinical practice. One formula bases dosage on mass of thyroid gland, absorbed dose, estimated uptake at time zero, and effective half-life (Teff) [8, 9]. Simplified formulas may include absorbed dose (determined by the physician), 24-hour radioactive iodine uptake (RAIU), thyroid weight, with/without a fixed Teff [10, 11].

Compared to the Teff, the measurement of a single 24-hour RAIU is convenient. However, several investigators [10–12] reported that rapid turnover (24-hour RAIU value is less than the 3- to 4-hour RAIU value) can be observed in 12%-32% patients with GD and that is a statistically significant predictor of radioiodine therapy (RIT) failure because of rapid clearance or turnover of ^131^I from the thyroid gland, with failure rates up to 55%[12].

Teff is one of the key factors for the success of RIT in GD [13]. It is commonly known that short Teff increases the risk for RIT failure, therefore, it is crucial for GD patients intended to receive RIT therapy to determine Teff. However, it is often argued that determining Teff is laborious, which requires no less than five patient visits over a 5-7 day period– this makes a novel, highly accurate Teff prediction model very valuable [14, 15].

In the present study, we investigated factors that could be used to predict Teff and further developed a Teff prediction model in our patient population.

## RESULTS

### Characteristics and correlation analysis between teff and continuous variables

The statistic characteristics of the continuous variables included age, disease duration, thyroid function indexes, thyroid autoantibodies, thyroid-stimulating hormone receptor antibody (TRAb) level, thyroid weight, and RAIU value at different time before RIT are listed in Table 1. Totally, there were 256 subjects (61 males and 195 females) involved in this study, 201 of whom were cured after a one-time ^131^I therapy and 55 of whom were unhealed and received the repeated therapy. The curative ratio of one-time ^131^I therapy was 78.5% and the incidence rate of hypothyroidism was 31.2%. Age ranged from 8 to 77 years with a mean of 39.41 ± 13.68 years, and the mean Teff of ^131^I actually determined was 5.80±1.45 days (range: 1.9-7.9 days) in our patient population. A correlation coefficient (r) was used to identify the relationship between Teff and related parameters. Since these indexes were abnormal distribution, the correlations of Teff with indexes of disease duration, thyroid-stimulating hormone (TSH), thyroglobulin antibody (TGAb), thyroid peroxidase antibody (TPOAb), TRAb, RAIU were analyzed by Spearman's test. As shown in Table 1, Teff correlated negatively with TPOAb, TRAb and thyroid weight (*P*<0.05, r = -0.191, -0.141 and -0.126, respectively), as well as positively with 24-hour, 48-hour, and 72-hour RAIU (*P*<0.05, r = 0.133, 0.341 and 0.442, respectively). However, we did not find any significant correlation between Teff and all the other parameters.

**Table 1:** Characteristics and correlation analysis between Teff and continuous variables in Graves’ disease patients

Parameters	Total (n=256)	Correlation coefficient (r)	*P* value
Age (years)	39.41±13.68*	-0.081	0.196
Disease duration (months)	20.0±57.0**	-0.023	0.710
FT_3_(pmol/L)	22.56±8.63*	-0.093	0.138
FT_4_(pmol/L)	69.87±38.54*	-0.055	0.379
TSH (μIU/mL)	0.006±0.005**	0.019	0.758
TGAb (IU/mL)	33.3±253.25**	-0.085	0.203
TPOAb (IU/mL)	378.0±938.50**	-0.191	**0.004**
TRAb (IU/L)	15.39±26.56**	-0.141	**0.027**
Thyroid weight (g)	38.16±25.17*	-0.126	**0.044**
24h-RAIU (%)	64.0±16.0** (28.7-87.3)†	0.133	**0.033**
48h-RAIU (%)	55.0±14.0**(26-83) †	0.341	**0.000**
72h-RAIU (%)	49±13.5**(18-77) †	0.442	**0.000**
Teff (days)	5.80±1.45*(1.9-7.9)†	1	-

* Data indicate mean ± standard deviation and are analyzed by Pearson's test.

**Data indicate median ± interquartile range and are analyzed by Spearman's test.

† Range.

FT_3_, free triiodothyronine; FT_4_, free thyroxine; TSH, thyroid-stimulating hormone; TGAb, thyroglobulin antibody; TPOAb, Thyroid peroxidase antibody; TRAb, TSH receptor antibody; RAIU, radioactive iodine uptake; Teff, effective half-life

### Comparison of categorical variables with Teff

**Figure 1:**
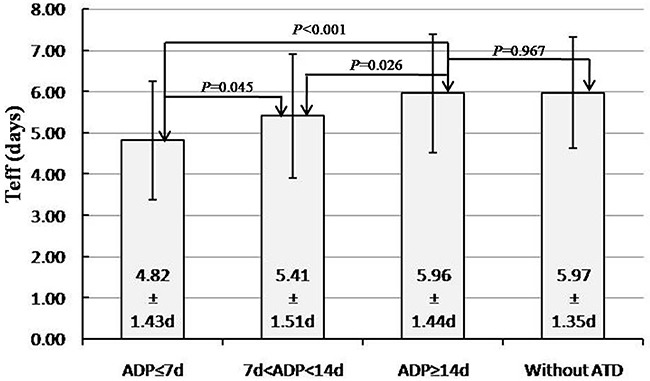
Qualitative and quantitative impact of anti-thyroid drugs discontinuation period on effective ^131^I half-life

As for the relationship between Teff and categorical variables, we performed 2-sided t test or one-way ANOVA analysis. The clinical data and statistical results are shown in Table 2. The results showed antithyroid drugs discontinuation period (ADP) was the only statistically significant factor (*P<*0.001, F=8.052). Furthermore, we found the mean Teff of patients with ADP ≥14d (5.96±1.44d) was significantly longer than that of patients with ADP ≤7d (4.82±1.43d) and 7d<ADP<14d (5.41±1.51d) (*P*<0.001, *P*=0.026, respectively) (Figure 1)- and a trend toward a longer Teff in the long ADP group than that in the short ADP group was evident, whereas the Teff before RIT did not differ significantly between patients with ADP ≥14d and those without ATD (5.97±1.35d) (*P*=0.967, Figure 1). Moreover, the Teff of patients with 7d<ADP<14d was also longer than that with ADP≤7d (*P*=0.045). The results revealed that longer ADP before RIT (especially ≥14d) led to a significantly longer Teff. However, statistics for male versus female and with ATD versus without ATD groups failed to reach significance (*P* = 0.817 and 0.372, respectively).

**Table 2:** Comparison of categorical variables in Teff

Parameters	Effective half-life	t/F value	*P* value
Sex			
Male (61)	5.77±1.51	0.232	0.817
Female (195)	5.81±1.43		
ATD or not			
With (201)	5.75±1.48	-0.893	0.372
Without (55)	5.96±1.35		
ADP‡			**0.000**
ADP≤7d (40)	4.82±1.43	8.052	1
7d< ADP<14d (51)	5.41±1.51		0.045
ADP ≥14d (110)	5.96±1.44		0.000
Without ATD (55)	5.97±1.35		0.000

‡ 55 patients (12 men and 43 women) did not receive antithyroid drugs.

ATD, anti-thyroid drugs;

ADP, ATD discontinuation period

### Multiple linear regression analysis

**Figure 2:**
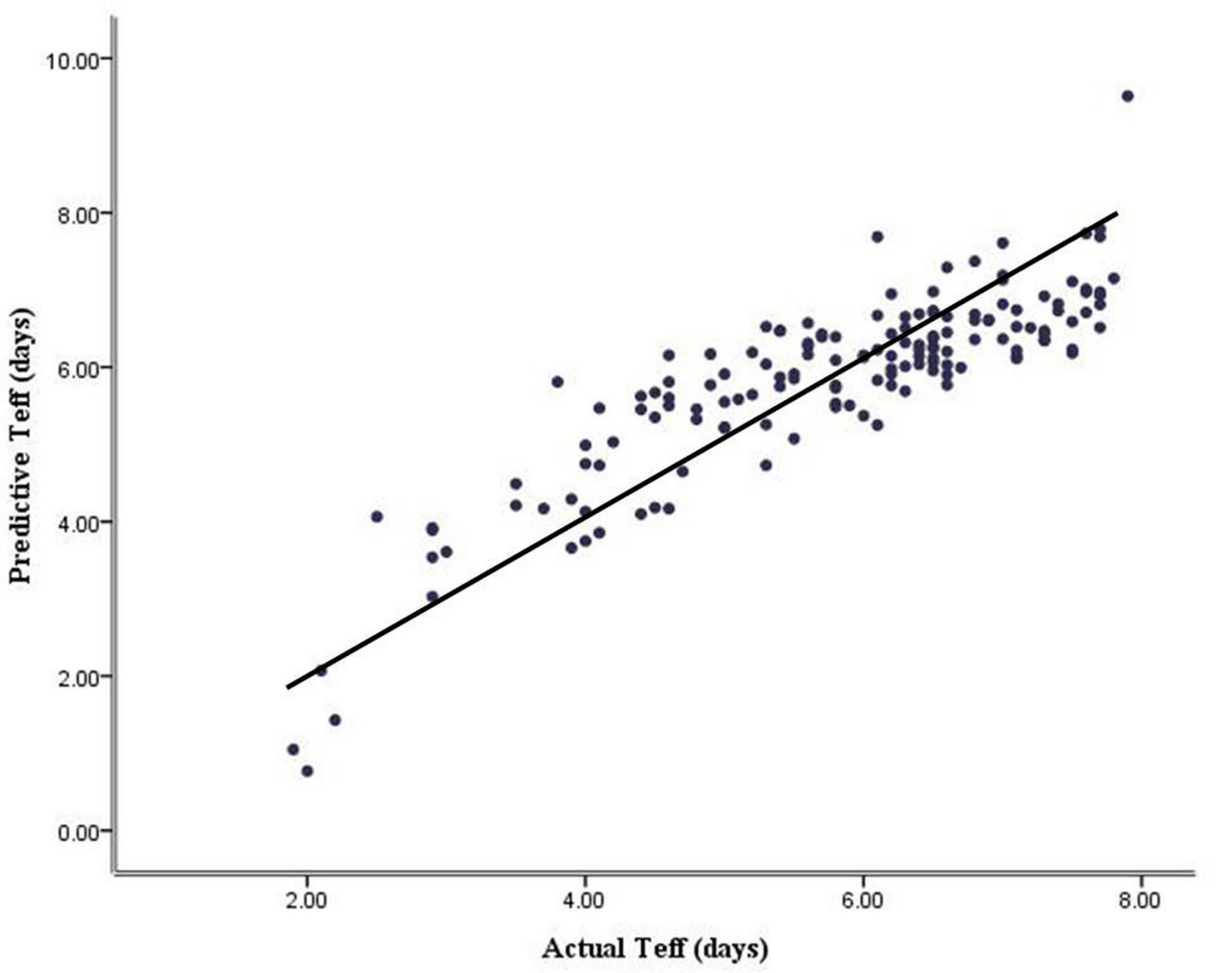
The relationship between predictive and actual effective ^131^I half-life analyzed by bivariate correlation (r = 0. 865, *P* < 0.001)

The effect of confounding factors was not taken into account during univariate analysis. Considering the independent variables related to each other, we applied a stepwise multiple linear regression (MLR) analysis to evaluate the factors which were independently correlated to Teff. Variables that were significant in the univariate analysis (seven factors) were entered into the stepwise method, which resulted in only two linear regression models, starting with 72h-RAIU, as shown in Table 3. MLR analysis revealed significantly independent influences of 24-hour RAIU (*P*<0.001) and 72-hour RAIU (*P*<0.001) on the Teff and these independent variables had also no collinearity between each other, as shown in Table 4. The MLR analysis yielded the following equation: predictive Teff=5.277+0.295×72-hour RAIU (%)-0.217×24-hour RAIU (%). Based on the regression equation, we could calculate the predictive Teff for all patients and further obtained a significantly positive correlation between predictive and actual Teff (r=0.865, *P*<0.001, Figure 2).

**Table 3:** Model summary of Teff based on linear regression analyses

Model	R	R Square	Adjusted R Square	Durbin-Watson
1	0.412^a^	0.169	0.162	
2	0.874^b^	0.764	0.760	1.948

a. Predictors: (Constant), 72h-RAIU

b. Predictors: (Constant), 72h-RAIU, 24h-RAIU

Dependent Variable:Teff

Teff, effective half-life

**Table 4:** Regression coefficients of MRL model based on the 7 independent variables

Independent variables	B coefficient (95%CI)	t value	*P* value	Collinearity statistics	
				Tolerance	VIF
Constant	5.277(4.598-5.956)	15.414	0.000		
72h-RAIU	0.295(0.264-0.327)	18.391	0.000	0.317	3.156
24h-RAIU	-0.217(−0.243--0.190)	-16.266	0.000	0.315	3.171

CI, confidence interval; RAIU, radioactive iodine uptake; VIF, variance inflation factor

## DISCUSSION

Effective thyroidal ^131^I half-life is one of the key factors for the success of RIT in GD [13]. It is commonly known that short Teff increases the risk for RIT failure, so it is crucial for GD patients intended to receive RIT therapy to determine Teff before RIT [16]. However, it is inconvenient to determine Teff directly, which demands ^131^I uptake measurements at 24 hours, 48 hours, and 4-6 days, requiring no less than five patient visits over a 5-7 day period, and some authors suggested that up to 10 measurements over a 7-day period are required [14, 15]. Based on this, we investigated the factors that could be used to predict Teff and further developed a Teff prediction model in our patient population, which was confirmed to be used more conveniently and practically for our routine work.

The present study demonstrated that the mean Teff determined during RIT was 5.80±1.45 days, ranges from 1.9 to 7.9 days in our patient population, which was approximately equivalent with previous study, [17] but higher than that reported by Kobe, C. et al. [14]. Using multivariate analysis, this study confirmed that 24-hour and 72-hour RAIU were the independent predictors that interfere with Teff of ^131^I during RIT of GD.

In the present study, univariate analysis showed that Teff had negative correlation with TRAb level, consistent with previous studies [4, 17]. Additionally, our study also revealed negative correlation between Teff and TPOAb and thyroid weight, whereas we found that Teff correlated positively with 24-hour RAIU, as well as 48-hour and 72-hour RAIU analyzed by Pearson's correlation analysis although the correlation was not strong enough (r=0.133, 0.341 and 0.442, respectively). Furthermore, a significant influence of ADP on the Teff was demonstrated. The results showed patients with longer ADP had significantly longer Teff, however, there was no significant difference on Teff between patients with ADP≥14d and subjects without ATD, indicating that ADP≥14d before RIT may be better for GD patients treated with ATD previously in order to obtain higher RIT success rates.

Previous studies revealed that antithyroid medication and thyroidal metabolic state could change Teff [4, 18]. However, using correlation analysis, our study did not find any correlation between Teff and free triiodothyronine (FT_3_), free thyroxine (FT_4_) level. Meanwhile, there was no significant differences found in the gender composition (*P*=0.817), and with or without ATD (*P*=0.372). The different results could at least partially be attributed to the selection bias of patient sources and different statistical analysis. Additionally, we speculate that different countries and ethnicities may also contribute to the differences.

Furthermore, we developed and tested a novel prediction model of Teff in this study. The related parameters that were significant in the univariate analysis were then included as independent variables for linear regression. MLR analysis showed that only 24-hour and 72-hour RAIU were related to Teff, and the remaining indexes TPOAb, TRAb, thyroid weight, 48-hour RAIU and ADP were excluded from the regression model even though they showed significant correlations with Teff during correlation or ANOVA analysis. A well-developed, convenient and highly effective prediction model validated for Teff poses a promising strategy for GD patients, with Pearson's correlation (r=0.865) between predictive and actual Teff.

### Limitations

This retrospective study had some shortcomings associated with this type of study. Please note that actually ADP is the period time defined from drug withdrawal to RAIU measurement. As the exact ADP of some patients could not be obtained, we divided the patients into 4 groups based on ADP. Additionally, we correlated continuous variables with Teff by bivariate correlation, but did not re-group them and compare the Teff among different groups. Moreover, there might be other factors we ignored affecting Teff. Therefore, larger prospective studies will need to be performed to confirm these preliminary results.

## MATERIALS AND METHODS

### Subjects

Two hundred and fifty-six GD patients (mean age 39.41±13.68; range, 8-77 years; 61 men and 195 women) treated for the first time with RIT at our institution between August 2014 and January 2016 were enrolled in this retrospective study. The therapeutic dose of ^131^I was calculated with the following formula: Dose (37 MBq) = estimated gland weight (g) × absorption dose (Gy/g) × 0.67/(Teff × max % uptake). Absorption dose = 100 Gy/g; 0.67 is a rectified factor. The patients receiving prednisolone before RIT, those who had received RIT before and cases with other etiologies for hyperthyroidism were excluded from this study. GD was diagnosed on the basis of thyrotoxicosis, increased thyroid hormones and decreased TSH, elevated thyroid radioiodine uptake, and diffuse goiter, or exophthalmos, or pretibial myxedema, or positive TRAb. We investigated the influences of age, gender, disease duration, thyroid weight, ATD or not, ADP, thyroid function indexes, thyroid autoantibodies, TRAb level and RAIU before RIT on Teff. This study was approved by the medical ethics research committee of Tianjin Medical University General Hospital and written informed consent was obtained from each patient.

For univariate and multivariate analyses, ADP was assigned as follows: ≤ 7 days, 7<ADP<14 days, ≥ 14 days, or without ATD administration. All patients with GD were scheduled to undergo 24-, 48- and 72-hour ^131^I uptake studies. Treatment with ATD such as methimazole and propylthiouracil was discontinued for at least 3 days before RAIU measurement, and dietary iodine restriction was initiated at least 1 week before RAIU measurement [19].

### Measurements

By chemiluminescent reaction principle, FT_3_ (reference 3.50–6.50 pmol/L), FT_4_ (reference 11.50–23.50 pmol/L) and TSH (reference 0.30–5.00μIU/mL) assays were performed on a fully automated ADVIA Centaur analyzer (Siemens Healthcare Diagnostics, New York, USA). TRAb (reference 0–1.50 IU/L) was measured by enzyme-linked immunosorbent assay (Medipan GmbH, Berlin, Germany). TgAb (reference 0–40.00 IU/mL) and TPOAb (reference 0–35.00 IU/mL) were also assessed by chemiluminescent reaction on a fully automated IMMULITE 2000 analyzer (Siemens Healthcare Diagnostics, Los Angeles, USA).

The thyroid volume was estimated by ultrasound (GE Vingmed Ultrasound Vivid Five, Horten, Norway) using the formula of a rotation ellipsoid [20]. The thyroid weight was calculated, assuming 1 ml to correspond to 1 g tissue. The thyroid ^131^I uptake value was measured after an oral tracer dose (approximately 74 kBq) of ^131^I through a radioactive iodine uptake probe (MN-6300XT Apparatus, Technological University, China), which could obtain the Teff [8]. The thyroid ^131^I uptake value was calculated using the following equation: RAIU (%) = (neck counts – background counts) × 100/(standard counts – background counts).

### Statistical analysis

Statistical analysis was performed using SPSS (Statistical Package for Social Sciences) 12.0 for windows (SPSS, Chicago, IL, USA). Normal distribution was tested with the Kolmogorov-Smirnov test. All data with normal distributions were expressed as mean ± standard deviation (SD) and data with non-normal distributions were expressed as median ± interquartile range (IQR). The relationships between Teff and continuous variables were analyzed by bivariate correlation (Pearson's or Spearman's correlation), while the relationships between gender, ATD or not, ADP and Teff were analyzed using 2-sided t test or one-way ANOVA analysis. Stepwise MLR analysis was performed to investigate factors that could be used to predict Teff, with a variable entrance criterion of 0.05 or less. All *P* values presented were two-tailed, and values < 0.05 were considered to be statistically significant.

## CONCLUSION

According to a single-factor analysis, Teff was related to TPOAb, TRAb, thyroid weight and RAIU (all *P*<0.05) and longer ADP (especially ≥14d) could lead to a significantly longer Teff. Stepwise MLR analysis indicated that only 24-hour and 72-hour RAIU were the independent variables related to Teff. The predictive Teff calculated based on the regression equation was significantly positively correlated with the actual one measured. The proposed model achieved higher prediction accuracy.

## References

[1] Burch HB, Burman KD, Cooper DS (2012). A 2011 survey of clinical practice patterns in the management of Graves’ disease. J Clin Endocrinol Metab.

[2] Smith TJ, Hegedus L (2016). Graves’ Disease. N Engl J Med.

[3] Ross DS (2011). Radioiodine therapy for hyperthyroidism. N Engl J Med.

[4] Moka D, Dietlein M, Schicha H (2002). Radioiodine therapy and thyrostatic drugs and iodine. Eur J Nucl Med Mol Imaging.

[5] Lind P (2002). Strategies of radioiodine therapy for Graves’ disease. Eur J Nucl Med Mol Imaging.

[6] Sisson JC, Avram AM, Rubello D, Gross MD (2007). Radioiodine treatment of hyperthyroidism: fixed or calculated doses; intelligent design or science?. Eur J Nucl Med Mol Imaging.

[7] Leslie WD, Ward L, Salamon EA, Ludwig S, Rowe RC, Cowden EA (2003). A randomized comparison of radioiodine doses in Graves’ hyperthyroidism. J Clin Endocrinol Metab.

[8] Willegaignon J, Sapienza MT, Coura Filho GB, Traino AC, Buchpiguel CA (2013). Determining thyroid (131)I effective half-life for the treatment planning of Graves’ disease. Med Phys.

[9] Wang R, Tan J, Zhang G, Zheng W, Li C (2017). Risk factors of hepatic dysfunction in patients with Graves’ hyperthyroidism and the efficacy of 131iodine treatment. Medicine (Baltimore).

[10] Morris LF, Waxman AD, Braunstein GD (2000). Accuracy considerations when using early (four- or six-hour) radioactive iodine uptake to predict twenty-four-hour values for radioactive iodine dosage in the treatment of Graves’ disease. Thyroid.

[11] van Isselt JW, Broekhuizen-de Gast HS (2010). The radioiodine turnover rate as a determinant of radioiodine treatment outcome in Graves’ disease. Hell J Nucl Med.

[12] Aktay R, Rezai K, Seabold JE, Bar RS, Kirchner PT (1996). Four- to twenty-four-hour uptake ratio: an index of rapid iodine-131 turnover in hyperthyroidism. J Nucl Med.

[13] Berg GE, Michanek AM, Holmberg EC, Fink M (1996). Iodine-131 treatment of hyperthyroidism: significance of effective half-life measurements. J Nucl Med.

[14] Kobe C, Eschner W, Wild M, Rahlff I, Sudbrock F, Schmidt M, Dietlein M, Schicha H (2010). Radioiodine therapy of benign thyroid disorders: what are the effective thyroidal half-life and uptake of 131I?. Nucl Med Commun.

[15] Furstner M, Hentschel M, Spanjol PM, Prenosil GA, Weidner S, Krause T, Klaeser B (2017). Technical Note: Determination of individual thyroid clearance effective half-life with a common handheld electronic dosimeter. Med Phys.

[16] de Jong JA, Verkooijen HM, Valk GD, Zelissen PM, de Keizer B (2013). High failure rates after (131)I therapy in Graves hyperthyroidism patients with large thyroid volumes, high iodine uptake, and high iodine turnover. Clin Nucl Med.

[17] Hautzel H, Pisar E, Yazdan-Doust N, Schott M, Beu M, Muller HW (2010). Qualitative and quantitative impact of protective glucocorticoid therapy on the effective 131I half-life in radioiodine therapy for Graves disease. J Nucl Med.

[18] Dunkelmann S, Kuenstner H, Nabavi E, Rohde B, Groth P, Schuemichen C (2007). Change in the intrathyroidal kinetics of radioiodine under continued and discontinued antithyroid medication in Graves’ disease. Eur J Nucl Med Mol Imaging.

[19] Kubota S, Ohye H, Yano G, Nishihara E, Kudo T, Ito M, Fukata S, Amino N, Kuma K, Miyauchi A (2006). Two-day thionamide withdrawal prior to radioiodine uptake sufficiently increases uptake and does not exacerbate hyperthyroidism compared to 7-day withdrawal in Graves’ disease. Endocr J.

[20] Perry RJ, Hollman AS, Wood AM, Donaldson MD (2002). Ultrasound of the thyroid gland in the newborn: normative data. Arch Dis Child Fetal Neonatal Ed.

